# Proceedings of the second annual dengue endgame summit: A call to action

**DOI:** 10.1371/journal.pntd.0013028

**Published:** 2025-04-28

**Authors:** Céline S.C. Hardy, Lauren E. Bahr, Alan L. Rothman, Kathryn B. Anderson, Giovanna Barba-Spaeth, Daniela Weiskopf, Eng Eong Ooi, Ernesto T.A. Marques, Mattia Bonsignori, Alan D.T. Barrett, Beth D. Kirkpatrick, Priscila M.S. Castanha, Marco Hamins-Puertolas, Rebecca C. Christofferson, George Dimopoulos, Fabiano Oliveira, Lillian W. Chiang, Albert I. Ko, Bhagwat Gunale, Prasad Kulkarni, T. Alex Perkins, Ilaria Dorigatti, Telisa Stewart, Jana Shaw, Michael A. Johansson, Stephen J. Thomas, Adam T. Waickman

**Affiliations:** 1 Department of Microbiology and Immunology, Upstate Medical University, Syracuse, New York, United States of America; 2 Department of Cell and Molecular Biology, Institute for Immunology and Informatics, University of Rhode Island, Kingston, Rhode Island, United States of America; 3 Unité de Virologie Structurale, Institut Pasteur, Université Paris Cité, CNRS UMR 3569, Paris, France; 4 Center for Vaccine Innovation, La Jolla Institute for Immunology, La Jolla, California, United States of America; 5 Program in Emerging Infectious Diseases, Duke-NUS Medical School, SingaporeSingapore; 6 Department of Infectious Diseases and Microbiology, School of Public Health, University of Pittsburgh, Pittsburgh, Pennsylvania, United States of America; 7 Translational Immunobiology Unit, Laboratory of Infectious Diseases, National Institute of Allergy and Infectious Diseases, National Institutes of Health, Bethesda, Maryland, United States of America; 8 Sealy Institute for Vaccine Sciences and Department of Pathology, University of Texas Medical Branch, Galveston, Texas, United States of America; 9 Department of Microbiology and Molecular Genetics, Larner College of Medicine, The University of Vermont, Burlington, Vermont, United States of America; 10 Department of Medicine, University of California, San Francisco, California, United States of America; 11 Department of Pathobiological Sciences, Louisiana School of Veterinary Medicine, Baton Rouge, Louisiana, United States of America; 12 Harry Feinstone Department of Molecular Microbiology and Immunology, Bloomberg School of Public Health, Johns Hopkins University, Baltimore, Maryland, United States of America; 13 Laboratory of Malaria and Vector Research, National Institute of Allergy and Infectious Diseases, National Institutes of Health, Bethesda, Maryland, United States of America; 14 Evrys Bio, Inc., Pennsylvania Biotechnology Center, Doylestown, Pennsylvania,; 15 Department of Epidemiology of Microbial Diseases, Yale School of Public Health, New Haven, Connecticut, United States of America; 16 Instituto Gonçalo Moniz, Fundação Oswaldo Cruz, Salvador, Brazil; 17 Serum Institute of India Pvt. Ltd., Hadapsar, Pune, Maharashtra, India; 18 Department of Biological Sciences, University of Notre Dame, Notre Dame, Indiana, United States of America; 19 MRC Centre for Global Infectious Disease Analysis and the Abdul Latif Jameel Institute for Disease and Emergency Analytics, School of Public Health, Imperial College London, London, United Kingdom; 20 Department of Public Health and Preventive Medicine, Upstate Medical University, Syracuse, New York, United States of America; 21 Department of Pediatrics, State University of New York Upstate Medical University, Syracuse, New York, United States of America; 22 Bouvé College of Health Sciences and Network Science Institute, Northeastern University, Boston, Massachusetts, United States of America; 23 Global Health Institute, State University of New York Upstate Medical University, Syracuse, New York, United States of America; University of Texas Medical Branch / Galveston National Laboratory, UNITED STATES OF AMERICA

## Abstract

On August 7–9, 2024, the second annual dengue “endgame” summit was held in Syracuse, NY, hosted by the Global Health Institute at SUNY Upstate Medical University. The meeting brought together attendees from around the world, with talks spanning healthcare, government control programs, basic research, and medical countermeasure development efforts. The summit goal was to work toward a better understanding of what dengue control could look like and the steps required to reach such a goal. The objectives of the meeting were to discuss the current global state of dengue, what dengue “control” might look like, and to discuss actionable pathways for achieving dengue control. Topics covered throughout the meeting included DENV immunity and pathogenesis, challenges in countermeasure development, innovative vector control strategies, dengue diagnostics, addressing challenges in science communication, and vaccine hesitancy. Several fundamental knowledge gaps were repeatedly highlighted by the summit attendees and were cited as critical barriers to the development, deployment, and evaluation of effective dengue countermeasures. These gaps include (1) the lack of a broadly applicable immunologic biomarker/correlate of DENV immunity and (2) the lack of universally accepted/applicable metrics for quantifying dengue severity in the setting of countermeasure evaluations. In addition, the lack of clear and consistent international leadership in the global dengue control effort was cited as a barrier to widespread and synergistic research and countermeasure development/deployment activities. Despite these persistent roadblocks, summit attendees expressed optimism that holistic and multi-tiered approaches—incorporating optimal use of existing and nascent countermeasure technologies deployed in collaboration with local communities—could be effective in progressing toward dengue control.

## 1. Introduction

On August 7–9, 2024, the SUNY Upstate Medical University Institute for Global Health hosted the second annual Dengue Endgame Summit. This meeting was hosted at the Syracuse University National Veterans Center, where 144 attendees representing 44 institutions and organizations from 10 countries (Belgium, Brazil, France, Jamaica, India, Singapore, Switzerland, Thailand, the United Kingdom, and the United States) convened. Since the 2023 Endgame summit [1], dengue cases have surged globally. Additionally, concerns regarding availability of key interventions and the developmental timeline of the next generation of DENV countermeasures have evoked uncertainty for the future of dengue control. This dynamic landscape of the dengue field motivated several of the topics covered in the 2024 meeting.

The title of the meeting was “The dengue endgame: a call to action”. This theme was inspired by the desire for the scientific, medical, and public health communities to unite and advance toward a future where dengue and other *Aedes*-borne diseases are concerns of the past. The format of the meeting aimed to encourage collaboration of a diverse range of speakers, moderators, and attendees from academia, government, biotech, pharma, and the medical community in addressing the meeting’s mission. This multidisciplinary and multisectoral gathering allowed the consideration of multiple unique and valuable perspectives on the challenges and opportunities associated with achieving sustained and functional dengue control. The summit was composed of 12 sessions with 2–4 themed presentations per session, a roundtable discussion, and a keynote presentation. Each of these sessions were followed by a moderated discussion and Q&A period, allowing for significant participant engagement with the session topics. The sessions were arranged such that they provided sufficient biologic and epidemiologic context for attendees from wide-ranging backgrounds. The structure of these proceedings recapitulates the meeting format: for each session, we describe an overview of the session objectives followed by highlights of the session and the discussion that followed each presentation. The key messages and specific calls for action are summarized in **Table 1**.

**Table 1:** The 2024 dengue endgame summit: key messages and specific calls for action.

Identifying immune markers associated with protection from dengue remains a challenge but is urgently needed to advance countermeasure development efforts.
A standardized, objective method for classifying dengue severity is needed to accelerate the evaluation of candidate DENV countermeasures.
Changing epidemiologic trends (e.g., increasing adult cases, severe primary infections) require revisiting assumptions about dengue immunopathogenesis.
Flavivirus immunity studies should extend beyond dengue-Zika interactions to assess the impact of preexisting immunity in target populations.
There is an urgent need to expand DENV surveillance with advanced serologic and molecular techniques to inform public health policy and countermeasure deployment strategies.
Effective health communication is essential to counter vaccine hesitancy and promote uptake of dengue countermeasures.
Dengue must regain priority as a global health issue, requiring interdisciplinary leadership to develop sustainable solutions.

## 2. Session 1: Reality check: Current state of dengue on the ground

The first session of the summit aimed to discuss and review the current state of dengue around the world to set the scene of past and current challenges in dengue control.

The first speaker of the event, **Jeremy Farrar**, DPhil (Chief Science Officer, WHO), welcomed attendees and gave a general update on the state of dengue from a global perspective. In his talk, Dr. Farrar stressed that the geographic impact of dengue is expanding, with transmission now routinely observed in Southern Europe, sub-Saharan Africa, and North America. While the overwhelming burden of dengue is still observed in Central and South America and Southeast Asia, factors such as increased urban population density, trade and travel, and climate change make the expansion of dengue one of the most significant infectious disease challenges of the 21st century. This expansion has resulted in the emergence of new epidemiologic and clinical profiles—such as infections in the elderly and/or individuals with co-morbidities—which complicate both the clinical management and social impact of dengue. Despite these challenges, Dr. Farrar emphasized the remarkable progress, which has been made in understanding dengue pathogenesis and in developing tools for the clinical management of dengue. Specifically, emerging tools such as molecular diagnostics and point-of-care rapid tests, immune modulators, and novel antivirals offer hope that the clinical impact of DENV infection may be manageable despite the ever-increasing infection rates. Additional strides have been made with novel vector control strategies such as the release of *Wolbachia*-infected mosquitos. Although an array of tools exists for DENV management, implementation of any one intervention in isolation is insufficient—there is no silver bullet for achieving dengue control. As with other infectious diseases, such as malaria and tuberculosis, a holistic approach strategically incorporating multiple interventions is necessary. These observations emphasize the need for collaborative work integrating tools for vector control, molecular diagnostics, and disease management for dengue control.

The second speaker of the session was **Laura Adams**, DVM, MPH (US CDC), who reviewed with the summit attendees the state of DENV in the United States (US). Dramatic DENV expansion has been evident in the Americas over the last decade, with the number of dengue cases reported as of August of 2024 already eclipsing the number of cases reported in the entirety of 2023, the previously highest number ever reported. While the majority of dengue cases reported in the contiguous US are travel-associated (94%), DENV transmission has been alarmingly high in several US territories including Puerto Rico (PR), American Samoa, and the US Virgin Islands (USVI). PR has been particularly hard-hit by emerging dengue cases, having declared a state of public health emergency on March 25, 2024, due to the first large-scale DENV outbreak since 2013. This emergence was coupled with a dramatic shift in the predominant DENV serotype from DENV-1 to DENV-3, with the most significant disease burden observed in individuals aged 10–19 years old, and more than half of the reported cases resulting in hospitalization. Dengvaxia—the only DENV vaccine currently available in PR—has faced several challenges to implementation, primarily due to low perceived risk posed by DENV, limited access to pre-vaccination serological screening (which is required for Dengvaxia administration), and pending discontinuation of production of the vaccine by the manufacturer. In addition to the DENV epidemic in PR in 2024, locally acquired cases have been reported in Hawaii, Florida, and Texas. These data highlight DENV as an emerging threat globally and in the US, and current and ongoing challenges with vaccine rollout which are concerning considering its global expansion.

### Key session insights and discussion points

- Despite advances in control measures, dengue is a growing global threat with drivers, such as increasing density of urban populations, trade and travel, and climate change which change and complicate the picture of dengue as it expands.
- Dengue is a growing problem in the U.S., with alarmingly high case numbers in endemic regions, such as PR, American Samoa, and USVI. There is also an increasing frequency of local transmission in states, such as Hawaii, Florida, and Texas, and a steady increase in the number of travel-associated cases throughout the rest of the country.

## 3. Session 2: Can we talk? Immunologic crosstalk between flaviviruses

The objective of the second session—moderated by **Alan Rothman**, MD (URI)—was to review and discuss our current understanding of the impact of immunologic crosstalk between flaviviruses as it pertains to DENV pathogenesis and countermeasure efficacy. This session was organized due to growing concern within the scientific and medical research communities regarding the potential for immunologic cross-talk between flavivirus in regions with active co-circulation of multiple flavivirus and/or established flavivirus vaccine programs.

**Kathryn Anderson** MD, PhD (SUNY Upstate) led the first portion of this session, discussing clinical and immunological impacts of heterologous flavivirus exposures on subsequent DENV infection. Epidemiological evidence of immune-mediated enhancement of secondary heterologous DENV infection has been extensively documented, with antibody-dependent enhancement (ADE) cited as the primary mechanistic explanation for the phenomenon [2–5]. However, the impact of this process for other flaviviruses with varying degrees of antigenic homology is still poorly understood [6]. Zika virus (ZIKV), Japanese encephalitis virus (JEV), and yellow fever virus (YFV) all exhibit significant genetic and antigenic similarity to DENV and overlap in geographic distribution. In addition, YFV and JEV vaccination is common in many parts of the world, including in regions with significant DENV endemicity, leaving open the possibility of vaccine-elicited immunologic cross-talk between DENV and other flaviviruses. Indeed, it has been observed that individuals with prior DENV exposure are less likely to develop clinically apparent YFV infections [7], and that individuals with immunity to DENV are less likely to be infected with ZIKV [8]. However, despite these observations which suggest that flavivirus cross-reactivity may play a protective role in the setting of heterologous viral infections, concern remains regarding the potential for disease enhancement. Notably, data from Thailand—where JEV vaccination is widespread—suggests that JEV seropositive individuals are more likely to be symptomatic when infected with DENV [9]. Furthermore, data have been published regarding the protective vs pathogenic role of DENV/ZIKV immunologic cross-talk, with data from Brazil suggesting that high levels of anti-DENV antibodies provide partial protection from subsequent ZIKV infection, while evidence from Nicaragua suggests that anti-ZIKV antibodies can increase an individual’s risk of severe dengue [10,11]. These results highlight the fact that the impact of preexisting flavivirus immunity on the clinical manifestation of a subsequent heterologous flavivirus infection is not bidirectional, and the sequence of flavivirus exposure can significantly impact the immunologic and clinical presentation of secondary infections.

The second speaker of the session, **Giovanna Barba Spaeth**, PhD (Institut Pasteur), presented on her group’s recent work on the impact of flavivirus immunologic cross-talk on vaccine immunogenicity. The foundational observation that inspired the analysis presented in Dr. Barbra Spaeth’s talk is that the primary antigenic target for IgG isotype antibodies elicited by a flavivirus infection is the fusion loop of the E protein, which is highly conserved across all flaviviruses [12]. Mature flaviviruses have a shared structure of 90 E protein dimers [13]. However, epitopes exposed during different stages of virus maturity differ due to dynamics of the E dimer [14,15]. Within flavivirus species, this results in a spectrum of antibodies generated in response to infection which vary with respect to functionality. Notably, antibodies formed in response to the fusion loop (FLE)—which are highly accessible in immature or partially mature virions—are generally poorly neutralizing and are thought to facilitate ADE [16,17]. In contrast, antibodies targeting E dimer epitopes (EDE) are able to effectively neutralize the virion [18]. In light of these observations, Dr. Spaeth discussed the results of her group’s recent study evaluating how preexisting flavivirus immunity shapes response to YF17D vaccination [19], where it was observed that individuals immunized with the purified-inactivated tick-borne encephalitis virus (TBEV) vaccine prior to YF17D vaccination exhibited higher titers of anti-YFV E protein IgG antibodies post-vaccination than those without TBEV vaccination. However, the higher titer of YFV-specific IgG observed in YF17D vaccinated individuals with baseline TBEV immunity was primarily directed toward the antigenically conserved and poorly-neutralizing FLE epitopes, exhibiting *in vitro* infection-enhancing activity against multiple flaviviruses (YFV, DENV, and ZIKV). Accordingly, preexisting immunity to flaviviruses (or at least immunity elicited by the purified-inactivated TBEV vaccine) skews vaccination response toward antigenically conserved epitopes, such as FLE. Altogether, this emphasizes the importance of deconstructing the polyclonal response to flaviviruses in endemic populations and considering the potential co-circulation of antigenically related viruses when selecting antigens for vaccine development.

Next, **Daniela Weiskopf**, PhD (LJI), discussed cross-reactivity between flaviviruses in the context of cell-mediated immunity (CMI). While immunologic cross-reactivity between flaviviruses has been extensively documented at the antibody level, much less is known about how sequential heterologous infections impact the development of flavivirus-specific cellular immunity. To this end, Dr. Weiskopf highlighted work illustrating that prior DENV exposures impact both the magnitude and specificity of T cell-mediated immunity elicited by a subsequent ZIKV infection, especially within the CD8 T cell compartment [20]. The immunologic basis for this was recently evaluated in cohorts from Nicaragua and Sri Lanka in DENV seropositive individuals probed with ZIKV-specific peptides. This analysis demonstrated a selective expansion of T cells specific for peptide sequences that were conserved between ZIKV and DENV, mirroring the selective expansion of B cells and antibodies specific for antigenically conserved B cell epitopes (such as FLE) that are observed at the antibody level following sequential DENV/ZIKV infection. In light of these results, Dr. Weiskopf discussed the importance of considering T cell cross-reactivity in the context of vaccine design, especially in populations with prior flavivirus exposure and immunity.

### Key session insights and discussion points

- The impact of non-DENV flavivirus-specific immunity on the risk of DENV infection or development of disease is complex and still incompletely understood.
- Immunity elicited by natural, non-DENV flavivirus infection may have a different impact on DENV infection risk than immunity elicited by vaccination, especially by purified/inactivated vaccines.
- The immunological outcomes of heterologous flavivirus exposures vary with the sequence of exposures and need not be bidirectional.
- Both humoral and cellular immune responses exhibit convergence toward antigenically conserved epitopes following sequential/heterotypic flavivirus infection/vaccination events.

## 4. Session 3: The changing face of dengue

The third session of the summit was chaired by **Kathryn Anderson** MD, PhD (SUNY Upstate) and held the objective of discussing the shifting clinical burden of dengue. While dengue has long been considered a pediatric illness—with the overwhelming burden of disease observed in children under 12 years of age—there has been an accelerating trend of DENV infections increasing in prevalence in older individuals, with significant implications in terms of clinical management and countermeasure deployment considerations.

The first speaker of this session, **Derek Cummings**, PhD (JHU), presented on the shifting clinical burden of dengue in an aging, warming world. Demographic changes in the human population including declining birth rates and increasing lifespan have impacted the incidence and age distribution of both DENV infection in general and severe dengue. While severe dengue—especially dengue hemorrhagic fever—has been historically considered a pediatric illness, increased vector control efforts and related public health strategies in many parts of the world have reduced the overall DENV force of infection sufficiently so that secondary DENV infection is experienced later in life than has historically been observed [21]. The frequency of severe dengue is increasing in aging individuals and bears important implications due to the presence of comorbidities at older age. This changing age distribution in combination with increased population mobility and diversity of viruses encountered have significantly impacted the force of DENV infection in many parts of the world [22]. Dr. Cummings postulated that the dengue pandemic is moving slowly toward equilibrium levels of immunity in the Western hemisphere, where changes in transmission patterns are measurable and predictable. Lastly, Dr. Cummings discussed the impact of climate change on dengue, where spatial changes in DENV distribution are being seen as a result of rising global temperatures, presumably due to the increasing range of mosquito vectors capable of transmitting the virus. However, periods of high temperatures are now frequently associated with an increase in both the frequency and size of severe DENV outbreaks many regions, with the brunt of the impact being felt in areas already experiencing endemic DENV transmission [23].

**Eng Eong Ooi,** MD, PhD (Duke-NUS) was the next speaker of the session, and discussed the impact of various external factors on dengue epidemiology and pathogenesis. Dr. Ooi started his presentation by highlighting the relevance of increasing obesity rates around the world to dengue pathogenesis. Prior analysis has also demonstrated that obese children are at a higher risk of developing severe dengue, as well as being at greater risk of infection overall [24]. This phenomenon is not unique to dengue, as obese individuals have also been identified as being more likely to have flu-like symptoms, albeit mild and short-lived, following YF vaccination. Using the YF vaccine that causes a mild, self-limiting viremic infection in human volunteers with written informed consent, his team found elevated baseline pro-inflammatory state in obese individuals, including cytokines such as IL-6 that have been previously associated with increased risk of severe dengue [25]. With further increase in plasma concentration of this and other pro-inflammatory cytokines after YF vaccine infection, the findings suggest potential targets for immunomodulatory approaches to prevent severe dengue in obese patients. As previously mentioned, changes in birth rates, lifespan, and countermeasure implementation have resulted in infection delays until later in life [21,22]. Dr. Ooi discussed the impact of these age-related shifts in dengue burden on vaccine implementation, where it is unclear what the efficacy of dengue vaccination might be in older adults. Developing vaccine strategies tailored to the unique immunologic profile of aging populations is of critical importance given both the shifts in severe disease risk, increased comorbidities, and immunosenescence. Hypo-responsiveness to vaccination and/or natural viral infection has been long considered a hallmark of an aging immune system [26]. Dr. Ooi stressed that appropriate vaccination strategies—especially those utilizing adjuvants such as AS01B—could overcome many of these barriers and still elicit durable and protective immunity [27]. Accordingly, developing vaccines that are specifically designed to retain activity in older individuals may fill a crucial gap in the current dengue countermeasure portfolio. Novel vaccines or the co-administration of “traditional” dengue vaccines alongside these next-generation vaccines may provide protection to an increasingly vulnerable and aging population.

### Key session insights and discussion points

- Reduced birth rates and increasing lifespan have impacted the distribution of severe dengue and bring to light considerations of DENV infection in older individuals.
- Rising global temperatures are impacting the geographic distribution of dengue and causing increased numbers of outbreaks in endemic areas.
- The effects of immunosenescence and durability of dengue immunity in an aging population need to be better understood -it is unclear whether immunity wanes considerably over time, and what dosing regimen should be used to vaccinate and sustain protection against dengue in older individuals.
- Obesity has been recently identified as a risk factor associated with symptomatic dengue infection and severe dengue in children, associations which require additional investigation.

## 5. Session 4: Signatures of DENV immunity & pathogenesis

The objective of the next session—moderated by **Eng Eong Ooi**, MD, PhD (Duke-NUS) was to discuss advances in our understanding of DENV pathogenesis and biomarkers of disease severity.

**Ernesto Marques**, MD (UPitt/Fiocruz) started the discussion by presenting on the dual role of the complement system in DENV infection. The complement system is composed of soluble and non-soluble factors and plays a role both in facilitating the lysis/clearance of infected cells and viral particles as well as controlling the inflammation elicited by apoptotic cells. Early studies examining the relationship between complement activation and dengue severity demonstrated greater rates of C3 and C1q consumption in severe dengue than in mild dengue, suggesting a role for the complement system driving dengue pathogenesis [28–30]. Since these early observations, additional work has pointed to a potential role for complement dysregulation or overactivation in DENV pathogenesis, thereby interfering with the functions of antigen-presenting cells (such as macrophages, dendritic cells, and monocytes), T cells, and B cells during the resolution phase of a DENV infection [30–32]. Components of the complement system have also been shown to directly interact with platelets to regulate vascular homeostasis. It has therefore also been hypothesized that complement activation molecules C3a and C5a may contribute to plasma leakage observed in severe dengue and the utility of complement-activation inhibitors as a host-directed dengue therapeutic. While not directly impacting the replication of the virus, this therapeutic strategy may provide a much-needed tool to address severe late-stage symptoms of DENV infections.

The second presentation of the session was by **Adam Waickman,** PhD (SUNY Upstate Medical University) who presented recent work demonstrating B cell receptor (BCR)-dependent enhancement of DENV infection. Classically, monocytes and macrophages were thought to be the main reservoir for DENV infection, especially during secondary dengue. However, a significant body of work has demonstrated that B cells are actually the most significant circulating reservoir of both DENV RNA and infectious virus during an acute DENV infection [33–36]. While B cells do not express conventional DENV entry receptors—such as DC-SIGN and the mannose receptor—they do express a membrane-bound immunoglobulin/B cell receptor (BCR). Recent work from Dr. Waickman’s group suggests that a potential mechanism for direct B cells is that DENV-specific BCRs are capable of facilitating both the binding and entry of DENV into permissive cells [37]. Normally non-susceptible cells, when transfected with a DENV-specific BCR are then susceptible to infection. Additionally, there is a selective and specific increase in the frequency of DENV-infectible B cells in PBMC from individuals who were previously flavivirus naïve after a primary DENV infection. BCR-dependent enhancement (BDE) of DENV infection represents a novel mechanism of immune-mediated enhancement of DENV infection that fills several gaps in current models of dengue immunopathogenesis.

Next, **Mattia Bonsignori**, MD (NIH) presented his group’s recent work on antibody and B cell cross-reactivity profiling in Rhesus macaques upon sequential ZIKV and DENV infections. The antigenic similarities between DENV and ZIKV have raised concerns for the potential of immune-mediated enhancement of infectivity in sequential DENV/ZIKV infections, analogous to what is observed during sequential heterotypic DENV infections. Dr. Bonsignori discussed key observations from a recent non-human primate (NHP) study which sought to define the impact of sequential DENV-2/-3 and/or ZIKV infections on the breadth and cross-reactivity of flavivirus-specific memory B cells generated in response to these repeated infections. Strikingly, prior DENV-2 and/or ZIKV infections had little-or-no impact on the virologic progression of a subsequent DENV-3 infection. However, sequential DENV-2/ZIKV infections were observed to elicit parallel DENV-3 cross-neutralizing responses instead of favoring convergence toward pan-neutralization. These findings highlight that the order and timing of sequential flavivirus exposures are important in shaping clonal antibody responses.

### Key session insights and discussion points

- The role of complement in dengue pathogenesis—and whether it is protective, or pathogenic—is unclear, and additional work is necessary to understand this to consider the use of complement inhibitors as a dengue therapeutic.
- BCR-dependent enhancement (BDE) is a novel mechanism of infection enhancement in dengue-specific B cells with implications to disease immunopathogenesis.
- Asymmetric ZIKV and DENV antibody responses at the clonal level may provide insight into immunogen design, assessing vaccine responses, and biomarkers of risk in natural infections.

## 6. Keynote presentation: Dengue: Confronting the pandemic

Day one concluded with a keynote presentation by **Scott Halstead**, MD, titled “Dengue: confronting the epidemic”, seeking to review and discuss both historic and contemporary challenges associated with DENV pathogenesis and the implications for achieving dengue control. Dr. Halstead began with a cautionary note, highlighting the record-breaking number of dengue cases reported in 2023 and 2024. In light of these trends and over 60 years of research and countermeasure development efforts, Dr. Halstead posed the question: “How are dengue cases still on the rise?” Dr. Halstead posited that a combination of inherent features of DENV immunopathogenesis (such as the phenomenon of immune-mediated enhancement of DENV infection), the emergence of ZIKV (the antigenic similarity of which may impact DENV immunity), and lack of effective vaccines or serosurveillance have posed significant challenges to dengue control.

Dr. Halstead next pivoted to review the history of dengue countermeasure development, highlighting the seminal observations that helped shape our current understanding of dengue. This includes the initial observations in the 1960s that the risk of severe dengue was highest in two very distinct groups of patients: (1) children and young adults experiencing their secondary/heterotypic DENV infection and (2) infants born to DENV-immune mothers. These critical observations laid the groundwork for the model of immune-mediated enhancement of DENV infection in general, and the concept of ADE in particular.

Inspired by these clinical observations, Dr. Halstead’s group pioneered experiments in Rhesus macaques in the early 1970s to dissect the mechanistic relationship between secondary/heterotypic DENV infection disease severity. These studies utilized newly isolated DENV 1–4 strains that were passaged in Primary dog kidney cells. By October 1979, two consecutive studies in macaques demonstrated that animals with preexisting anti-DENV immunity had significantly elevated viremia when compared with naïve monkeys [38–40] when challenged with a heterologous DENV serotype, consistent with the virologic profiles observed in patients experiencing secondary dengue. Furthermore, these studies described for the first time the phenomenon of ADE—the failure of IgG antibodies to effectively neutralize and instead, increase infection in monocytes/macrophages [39]. Dr. Halstead emphasized how these observations in NHPs provided a mechanistic explanation for the increased risk of severe disease seen in both infants born to DENV-immune mothers and children experiencing their second DENV infection. In both cases, Dr. Halstead posited that the greatest risk of severe disease will be seen when anti-DENV antibodies wane to sub-neutralizing levels, be those antibodies derived from a DENV-immune mother (in the case of the infants) or as the result of a primary DENV infection.

Next, Dr. Halstead discussed observations pertaining to the 2015/2016 ZIKV outbreak in Brazil, where it was observed that dengue cases plummeted in the years immediately following the emergence/reemergence of ZIKV in the region. However, this respite from dengue was short-lived, as this lull in cases was followed by a dramatic resurgence in DENV transmission. While the mechanisms responsible for these dynamic transmission cycles are complex and still incompletely understood, Dr. Halstead highlighted the possibility that ZIKV/DENV immunologic cross-reactivity may have played a role both in the suppression of DENV transmission immediately after the ZIKV outbreak (due to high levels of cross-reactive antibodies) as well as the reemergence of DENV several years later (due to immune-mediated enhancement).

In his closing remarks, Dr. Halstead reemphasized that the dengue epidemic is still on the rise despite years of advances in our understanding of DENV pathogenesis. Many of the challenges faced today parallel initial historic challenges due to the complexity of pan-flavivirus interactions and the risk of disease enhancement. Dr. Halstead concluded that in order to solve the dengue problem, we need to think critically about gaps between the bench and epidemiologic work and redirect research to address important and clinically tractable problems.

## 7. Session 5: Advances in preclinical & clinical models for assessing dengue countermeasures

The objective of the session, moderated by **Natalie Collins,** PhD, MPH, MLS (WRAIR), was to discuss advances in models to assess or predict dengue countermeasure efficacy and how these tools can support regulatory decisions relating to countermeasure deployment.

To begin the session, **Kirk Prutzman,** PhD (FDA) provided an overview of the regulatory considerations associated with preclinical and clinical models for dengue vaccine evaluation. He provided an overview of the FDA vaccine review and approval process, highlighting the specific challenges associated with conducting dengue vaccine studies and specific FDA requirements of a Phase III dengue vaccine trial. As with all trials involving biologics, Dr. Prutzman emphasized that licensure requires data demonstrating that a vaccine is safe, pure, and potent. The potency or effectiveness of any vaccine must be demonstrated by an adequate and well-controlled phase 3 clinical trial measuring protection against disease. In dengue, this is particularly challenging given that there are currently no established immunologic markers of dengue protection; thus, endpoints of clinical dengue disease are used. Further challenges are faced due to the possibility of enhanced disease in response to vaccination. This requires the collection of longitudinal data to demonstrate safety against vaccine-induced enhanced disease by an adequate and well-controlled clinical trial. Long-term follow-up and safety data are thus required by the FDA, making dengue vaccine trials a long, difficult, and expensive process. Dr. Prutzman posited that one key route toward accelerating the licensure of next-generation DENV vaccine would be the identification and validation of early biomarkers of DENV vaccine efficacy and safety.

Next, **Alan Barrett, PhD (**UTMB) discussed the contribution of preclinical animal models for assessing and down-selecting candidate dengue countermeasures. Despite DENV being an obligate primate pathogen, a number of mouse models have been developed and used to evaluate candidate DENV countermeasures [41]. The most commonly used small animal model of DENV infection is currently the AG129 mouse, an IFNα/β/γ receptor-deficient model that has been shown to be highly susceptible to multiple strains of all four DENV serotypes without requiring mouse adaptation of the viruses [42,43]. Although DENV infections are generally lethal in this model—which differs from the normal clinical progression of DENV in its natural human/primate host—the AG129 model has proven to be a valuable tool for down-selecting early-stage countermeasures prior to evaluation in non-human primates and/or human subjects. In addition to mice, Dr. Barrett highlighted several additional animal models of DENV infection which have been used to evaluate DENV vaccines and therapeutics, including swine [44], tree shrew [45], and several species of non-human primates [46]. While swine and tree shrew are currently still discovery models, multiple NHP models of DENV infection have been rigorously evaluated and extensively utilized for vaccine evaluation. Most notably, this includes Rhesus and Cynomolgus macaques, which are still considered the gold-standard NHP model for evaluating DENV countermeasures prior to assessment in human subjects. However, the clinical presentation of DENV infection in all NHP models is very mild, meaning that most studies have to rely on reductions in viremia as the sole readout of the potential of an intervention to have a clinical benefit. No current animal model accurately recapitulates human disease or the complexity of the immunologic process following DENV infection, meaning that despite the advances in animal models for DENV infection human subject studies are still required to assess the safety profile and potential for clinical benefit of any candidate DENV countermeasure.

The next speaker of the session, **Stephen Thomas, MD** (Director, Global Health Institute, SUNY Upstate) gave a presentation entitled “*Lessons Learned from the Dengue Human Infection Model Consortium*”. Dr. Thomas started his presentation by articulating that the goal of experimental human dengue infection is to safely and consistently recapitulate the clinical, virologic and immunologic events which occur following natural primary DENV infection. There are multiple applications for such a model, ranging from providing an unparalleled opportunity to understand the early virologic and immunologic features of a primary DENV infection and extending to DENV countermeasure and diagnostic development. To this end, the Dengue Human Infection Model (DHIM) consortium has developed and evaluated three human challenge virus strains: DHIM-1, DHIM-3, and DHIM-4 [47–51]. In these models, under-attenuated DENV-1, -3, and -4 viruses—all derived from live-attenuated vaccine candidates that were deemed safe but too reactogenic for additional clinical development—produce mild to moderate dengue illness in subjects, with corresponding clinical, laboratory, virologic, and immunologic correlates. Throughout these challenge studies, each serotype has demonstrated slightly unique replication kinetics, with the DENV-4 exhibiting the fastest onset of RNAemia, followed by DENV-3, and finally DENV-1. Dr. Thomas discussed the results of a recent prime-boost vaccination study where individuals were vaccinated with a tetravalent DENV vaccine, followed by DENV-1 challenge [47]. Interestingly, the majority of vaccinees were not protected from DENV-1 challenge and exhibited accelerated dengue virus replication relative to unvaccinated participants who were challenged with DENV-1. This suggests a potential vaccine-induced immunologic enhancement (accelerated viremia) of the challenge infection. Dr. Thomas also discussed some preliminary results of a current homologous rechallenge study, which has demonstrated that protection persists over years and that cellular immune responses are measurable in the context of no detectable viremia.

This session’s final speaker**, Beth Kirkpatrick, MD** (UVM) presented work which leveraged DENV-2 and DENV-3 Controlled Human Infection Models (CHIMs) developed by the teams at the NIH, UVM, and Johns Hopkins to evaluate vaccines and antiviral agents. Similar to the DHIM studies described by Dr. Thomas, these “NIH models” have been used for over a decade [52–54]. The NIH DENV-2 and DENV-3 CHIM studies are powerful tools dissecting fundamental features of DENV pathogenesis as well as assessing the potential efficacy of candidate dengue countermeasures in a safe and controlled fashion. Challenge strains have been developed for DENV-2 and DENV-3 and have been shown to be capable of inducing predictable, low-level viremia, short-lived cytopenia, and mild-moderate rash in participants [52–54]. These challenge strains have been leveraged to test the efficacy of several candidate DENV vaccines, including the tetravalent NIH-developed TV003 and TV005 vaccines, and an experimental DENV-1, -3, -4 trivalent vaccine formulation [52,53]. The TV003 and TV005 vaccines were developed by the NIH and are now licensed and currently in late-stage clinical development by several companies, including the Instituto Butantan [55], Merck vaccines [56], the Serum Institute of India [57]. This work demonstrated that TV003 vaccination provided 100% protection from DENV2 challenge at 6 months, while TV005 demonstrated 100% efficacy against DENV-2 or DENV-3 challenge [52,53]. However, trivalent vaccination (components of DENV-1,3,4) was insufficient to provide full protection following DENV-2 challenge, demonstrating the importance of vaccine replication in the setting of multivalent live-attenuated vaccine platforms. In addition, Dr. Kirkpatrick highlighted the recent use of these models to evaluate the efficacy of the JNJ-1802 antiviral (Johnson & Johnson) following DENV-3 challenge. This work demonstrated that antiviral administration significantly reduced viral load relative to individuals receiving a placebo prior to challenge, and a dose-dependent antiviral effect on detectability and time of onset of DENV-3 RNAemia. Additionally, no IgM/IgG seroconversion occurred in half of the inoculated subjects who had no detectable DENV RNA. A number of trials are on the horizon using the CHIM models for evaluating various vaccine and anti-viral candidates. Finally, the first use of these CHIM in an endemic country is ongoing in Bangladesh, using the DENV-2 CHIM to test tetravalent vaccine efficacy with challenges performed at 6, 12, and 24 months after vaccination.

### Key session insights and discussion points

- Phase 3 efficacy trials of tetravalent DENV vaccines are difficult and lengthy to conduct, since clinical dengue infection is used as the endpoint. Better biomarkers of response, efficacy, and safety would accelerate next-generation dengue vaccines.
- A number of animal models have provided insight into DENV pathogenesis, however, none of these entirely recapitulate features of human disease, and better models are needed.
- Human infection models provide a useful tool for modeling human DENV infection and providing key insights into disease pathogenesis and human immunity.
- Human infection models recapitulate human infection and uncomplicated disease with DENV and further serve as invaluable tools for assessing novel vaccine and anti-viral agent efficacy.

## 8. Session 6: Advances in dengue diagnostics and risk prediction: implications for dengue mitigation and management

The sixth session of the summit was moderated by **Alan Landay, PhD** (UTMB), and aimed to review recent advances in dengue diagnostics and surveillance technology and implications for countermeasure deployment.

The session started with a presentation by **Frank Middleton, PhD** (SUNY Upstate), who spoke regarding the performance of an optimized saliva-based nucleic acid detection and quantification test for DENV infection. Dr. Middleton started by highlighting the fact that the oral cavity in humans is a rich site of immune activity, making saliva an outstanding biospecimen for detecting immune signals following viral infections. While saliva-based PCR assays were widely used during the SARS-CoV-2 pandemic, they have yet to be as widely utilized for the detection of other, non-respiratory pathogens. Indeed, while prior studies have shown that DENV RNA is present in saliva, its abundance was shown to be significantly lower than the levels observed in serum [58]. In spite of this technical challenge, Dr. Middleton stressed that the advantages of saliva-based diagnostics—including the noninvasive nature of sample collection, the ability for individuals to self-collect, and the ability to more easily collect samples from young children—justified evaluating the approach in a rigorous fashion. Accordingly, he sought to develop a multiplexed, qRT-PCR assay capable of detecting and differentiating all four DENV serotypes in saliva. The performance of the multiplex assay on cell culture supernatants had 100% sensitivity, high signal compatibility, and zero cross-channel artifacts. When applied to saliva samples from a recent DENV-1 human challenge study, the assay was capable of detecting DENV-1 in all infected human challenge subjects who were also positive by conventional serum-based PCR. Moving forward, the group plans to develop loop-mediated isothermal amplification (LAMP) assays, which utilize an isothermic reaction producing colorimetric results to allow more rapid point-of-care testing. Additional considerations include whether biomarkers of host response may be detectable in the saliva, where some preliminary work comparing blood and saliva profiles has shown correlates in interferon signaling and host–virus interactions.

Next, **Priscila Da Silva Castanha,** PhD, MPH (University of Pittsburgh) presented on the discovery of biomarkers via epitope surrogate technology, leading to the development of a novel assay that allowed for the differential serodiagnosis of ZIKV and DENV infections. This work leveraged the antigenically agnostic approach for biomarker identification of Peptoid-Inspired Conformationally Constrained Oligomers (PICCOs) [59]. The PICCOs combinatorial library consists of small synthetic molecules that mimic a wide variety of natural epitopes. By down-selecting from over half a million molecular candidates contained within this array of synthetic, non-natural oligomers, the method was able to identify a lead candidate “CZV1-1” that mimicked very specific and sensitive ZIKV epitopes without DENV cross-reactivity [60]. In addition, Dr. Castanha described a methodology to characterize the native antigen that these epitopes were mimicking and their correlates with antibody function. “CZV1-1” mimicked a nonlinear, neutralizing conformational epitope on EDIII of ZIKV [60]. Dr. Castanha highlighted that understanding which epitopes are less likely to elicit cross-reactive antibody responses using this methodology is an important consideration for optimizing epitopes used as vaccine immunogens, which may be applicable for ZIKV or DENV.

Next, **Marco Hamins-Puertolas,** PhD (UCSF) discussed work leveraging serological data to infer dengue virus infection and quantify risk. Most studies focus on the study of symptomatic dengue, however, the majority of DENV infections are subclinical, representing a gap in the field. Dr. Hamins-Puertolas presented work which aimed to develop a framework for using serologic data to identify subclinical infections. Hemagglutination inhibition assay (HAI), Enzyme immunoassay (EIA), and PCR assays were applied to identify infections in paired acute and convalescent samples from the Kamphaeng Phet Cohort Study (KFCS) representing longitudinal multi-generational household samples [61,62]. In this work, comprehensive serologic data allowed for the more accurate identification of subclinical infections than any individual assay alone. However, importantly, this prediction was context dependent, where the assays available and timing between sample collection were relevant. These types of methods, capable of identifying subclinical infections, allow for the estimation of less biased correlates of protection. In particular, evidence from this same cohort suggests herd immunity may act at the household level, that low preexisting antibodies were associated with an increased probability of symptomatic infection, and sex and age were also found to impact probability of symptomatic infection which requires additional investigation.

### Key session insights and discussion points

- Diagnostic tests using saliva samples may provide more rapid, easily accessible diagnostics with potential to build models to understand DENV immunopathogenesis.
- Adaptation of the PICCOs assay for other biologic samples and other pathogens may provide insight into epitopes relevant for risk mitigation and vaccine design considerations.
- Combining data from multiple serologic assays provides the best prediction of past subclinical infections within an infected household, and the associations of age and sex with likelihood of symptomatic infection requires further investigation.

## 9. Session 7: Vectors, vector control, and vector-directed interventions

The next session, chaired by **Saravanan Thangamani,** PhD (SUNY Upstate), had the objective to discuss and review the current state of vector control technology and vector-directed interventions and their implication for dengue control.

**Rebecca Christofferson**, PhD MApSt (LSU), began by giving an overview of the history of vector control and a look at where vector control stands now from a viral perspective. Disease has long been associated with flying insects and thus measures to control vectors of disease have evolved over many decades. These practices have included attempts at breeding reduction to reduce the transmission of YFV in the 1920s and the rollout of DDT across Brazil and the Western Hemisphere in the 1940s. Used throughout the 70s and into the 80s, DDT was successful in partially eradicating vectors throughout South America but was restricted and subsequently banned in the 1990s due to environmental impacts. Currently, many methods of vector control are in use with varying degrees of efficacy and risk, from insecticide spraying of domiciles and spatial repellents to symbionts used in blocking infection in mosquitoes such as *Wolbachia* and *Rosenbergiella* sp. Each intervention comes with their own challenges such as scalability, labor force, and public acceptance. For example, the roll out of genetically modified mosquitoes has met with some resistance from the public and could confound future efforts to scale up and expand these efforts. From a viral perspective, Dr. Christofferson points us to a more systemic approach for effective vector control combining vector population control, suppression, and changes in human behaviors to best combat arboviral disease.

The second talk of the session focused on a topic touched on in Dr. Christofferson’s presentation, genetically modified mosquitoes as a means for disease control. **George Dimopoulos**, PhD (JHU), presented his work on exploiting existing pathways of innate immunity in *Aedes* mosquitoes to block transmission of DENV and other arboviruses [63,64]. Targeting the RNAi pathway in *Aedes* provided pan-antiviral activity, whereas targeting the JAK-STAT pathway only provided resistance to DENV. Use of a bloodmeal inducible promotor to overexpress transgenes Dicer2 and R2D2 resulted in mosquitoes with a significantly greater resistance to DENV, CHIKV, and ZIKV without impacting adult fitness [64]. When AGO2 was knocked out the genetically modified mosquitoes became susceptible to fatal viral infection. DNA repair and autophagy pathways were impaired in the AGO2 KO mosquitoes, indicating that overexpressing or knocking out genes in the in the siRNA pathway can alter the outcome of viral transmission in the mosquito vector.

For the final talk of this session, **Fabiano Oliveira**, MD PhD (NIH), spoke about ongoing work on how the human responses to mosquito saliva can impact the outcome of DENV infections. It is known that mosquito saliva is immunogenic and enhances arboviral infections in mouse models. AeD7L1+2 salivary protein was determined to be a better biomarker of *Aedes* exposure than whole salivary gland extract or AeD7L1 or 2 alone [65]. Using a cohort study based in Cambodia samples were taken from children with asymptomatic and symptomatic DENV infection. Children with asymptomatic DENV infection had higher anti-AeD7L1+2 titers than those experiencing a symptomatic DENV infection [65]. Studies are also being done to better understand the immune response to mosquito salivary proteins in human skin after a bite. After a controlled mosquito feeding, biopsies of skin from the feeding site demonstrated cellular infiltration to the bite site 48 hours post bite. A DENV-Infected Mosquito Controlled Human Infection Model (dimCHIM) is in development to further test the effects of mosquito salivary protein on DENV transmission and outcome.

### Key session insights and discussion points

- Vector control will require an integrated and multifaceted approach.
- Science communication will play a role in effective vector control, especially for strategies utilizing genetically modified organisms.
- The innate immune pathways of mosquitoes can be exploited to prevent or enhance viral replication within the vector and may present an opportunity to interrupt viral dissemination if appropriately targeted.
- Mosquito salivary proteins are immunogenic and can be used as biomarkers of exposure.
- Mosquito salivary proteins may also be playing a role in disease outcome, with further models to investigate these mechanisms being developed.

## 10. Session 8: Next-generation countermeasures

The eighth session of the summit -moderated by **Aaron Farmer,** DO, MPH (AFRIMS)- aimed to review and discuss the state of next-generation dengue countermeasures.

First, **Lillian Chiang,** PhD (Evrys Bio) presented on the potential for host-directed antiviral compounds to fill existing gaps in dengue countermeasure portfolios. While the overwhelming majority of clinically-approved antiviral therapies directly target the infecting pathogen (direct-acting antivirals), Dr. Chiang posited that broad-spectrum, host-targeted antivirals can play a critical role in supporting pandemic preparedness efforts and can effectively synergize with traditional countermeasure strategies. While there is a long history of targeting host factors to improve antiviral responses, Evrys Bio has focused development efforts compounds that modulate Sirtuin 2 (SIRT2) activity. SIRT2 is a class 2 lysine deacetylase and regulator of transcription that acts to post-translationally modify cellular processes required for productive viral infections [66]. Evrys Bio recently developed a small molecule allosteric inhibitor of SIRT2 as a prospective broad-spectrum/host-directed antiviral compound [67]. Thus far, SIRT2 inhibition has been shown to exhibit broad antiviral activity, showing both *in vitro* as well as *in vivo* efficacy against both DNA and RNA viruses, including flaviviruses Zika and DENV-2. Notably, due to its mechanism of action, SIRT2 inhibition results in very low rates of viral drug resistance in serial passaging. Mechanistically, SIRT2 inhibition also acts to reactivate cellular p53, which is frequently modulated by viruses to enhance cell survival, thereby resulting in death of infected cells [68]. Given its broad-spectrum activity potential and efficacy in similar virus families, work is planned to translationally assess the utility of SIRT2 modulators in DENV infection.

Next, **Phil Santangelo,** PhD (Emory/Georgia Tech) presented on his team’s recent work developing and optimizing a treatment for DENV infections leveraging mRNA-encoded Cas13 proteins co-delivered via lipid nanoparticles with DENV-specific guide RNAs. Cas13 is an RNAase, which can rapidly and effectively degrade RNA molecules when targeted with an appropriate guide RNA [69]. Cas13-mediated degradation of viral RNA has previously been shown to be effective in preventing and/or treating SARS-CoV-2 and influenza virus infection both in vitro and in vivo [70], and Dr. Santangelo’s team decided to build upon these results by assessing the ability of Cas13 to target and degrade the RNA genome of DENV. To this end, his team developed a guide RNA sequence that targeted a highly conserved region of DENV NS5, which was co-delivered with Cas13 mRNA via Lipid Nanoparticle (LNP) [71]. This strategy showed promising efficacy in mitigating DENV-2 infection in vitro without altering cell survival, and DENV-3 infection in AG129 mice after a single dose administered one day post-infection. Further, treatment with mRNA encoded Cas13 and/or its guide demonstrated no alterations to cell viability or induction of cell toxicity. This work is currently moving toward testing this methodology in DENV-4 in vivo models and characterization of DENV-2 infection in NHP models.

### Key session insights and discussion points

- Host-directed antivirals offer the possibility to provide broad antiviral activity in the setting of DENV infection while synergizing with vaccination and/or direct-acting antivirals.
- Novel genetic tools—such as Cas13—offer the opportunity to directly target the DENV genome for degradation. However, this is still an early-stage technology and questions remain regarding the scalability and deployability of the approach.
- It is still unclear how the use of antivirals may impact the development of DENV-specific adaptive immunity when used after the onset of infection.

## 11. Session 9: Advanced countermeasure update

The objective of the next session—moderated by **Albert Ko,** MD (Yale)—was to review and discuss the deployment and evaluation of dengue vaccines and therapeutics currently in clinical development.

First, **Shibadas Biswal,** MBBS, MD (Takeda) presented updates on Takeda’s dengue vaccine and post-licensure plan. TAK-003 (Qdenga) was designed to meet the complex challenges of dengue vaccination and has been evaluated in a comprehensive clinical development program [72]. In these studies, the tetravalent immune response for TAK-003 has been comprehensively studied, demonstrating an engaged innate immune response signature, eliciting neutralizing antibodies to all four DENV serotypes, and stimulating multifunctional T cell responses. Further, this vaccine has demonstrated a balanced benefit/risk safety profile, and co-administration studies with vaccines for Hepatitis A [73], YFV [74], and human papilloma viruses have been performed with comparable tolerability to administration alone. As of now, over 30 dengue endemic and non-endemic countries have registered the vaccine for use irrespective of serostatus. Ongoing clinical development and real-world evidence studies will help address knowledge gaps.

The next speaker of the session **Bhagwat Gunale,** MD (SII)- provided an update on a Phase 2 clinical trial of a dengue monoclonal antibody (mAb). Dengue mAb (VIS513) targets a conserved region of the EDIII protein, conserved across all four serotypes [75]. VIS513 has demonstrated potent neutralization of all four DENV serotypes across several genotypes. In a humanized mouse model, Dengue mAb protected animals challenged with a lethal dose of DENV, mitigating thrombocytopenia, vascular leakage, and reducing viremia [75]. In Phase 2 trial, dengue mAb treatment by intravenous infusion significantly decreased viral load at 24 hours, and demonstrated significant reduction in time to clearance of fever, with a trend toward rapid recovery of platelets in dengue mAb treated groups. Moving forward, the group is performing a Phase 3 study evaluating the efficacy and safety of VIS513 in children and adults.

**Guillermo Herrera-Taracena,** MD, MBA (Johnson & Johnson) then gave a talk entitled “*Latest Insights from a Phase 2 Dengue Prophylactic study with JNJ-1802 -Mosnodenvir: An Overview of Preliminary Baseline Data*”. JNJ-64281802 (JNJ-1802, Mosnodenvir) is a small molecule inhibitor of DENV NS4B which has shown promising anti-viral potential [76,77]. A phase 2 randomized, double-blind, placebo-controlled, multicenter interventional study assessed the efficacy and safety of two dose regimens of JNJ-1802. This study was conducted to test the prevention of DENV infection in household contacts (HHCs) of a laboratory-confirmed DENV-infected index case. This study is currently being conducted in over 10 countries. Despite intensive sampling schedules, the study has been able to achieve its operational goals (90% dosing completion and 88% study completion) in terms of recruitment and retention. Dr. Herrera commented on how communication is key to enrollment in these studies, where flexibility, openness, established relationships with community structures, and a humble approach to community members are big contributors to success. These studies provide unique and meaningful data by capturing comprehensive baseline status and characteristics with extensive sampling, providing novel insight into the potential to understand dengue dynamics in the community and individual. Further, samples collected from these studies will lend insight to the circulation of the four DENV serotypes across geographical regions.

### Key session insights and discussion points

- The evaluation and deployment of next generation DENV countermeasures is complex and requires careful study design and execution.
- The success of novel therapeutics for dengue will depend on clear delineation of the population groups that will benefit from this intervention and local infrastructure to detect DENV infection in a timely diagnosis
- The identification of small molecule inhibitors of DENV function and mAbs offer a novel strategy for pre-exposure prophylaxis in the prevention of dengue in high risk population groups and outbreak settings, but evaluation of these new countermeasures is complex and requires careful study design and execution

## 12. Session 10: Challenges and opportunities in dengue countermeasure development and deployment during a dengue epidemic

The objective of session 10 of the summit was to discuss how the current global dengue situation—characterized by unprecedented dengue outbreaks both in traditionally endemic regions as well as regions that have not traditionally seen sustained DENV transmission—has impacted DENV countermeasure development efforts. This session was moderated by **Stephen Thomas,** MD (SUNY Upstate), and the discussion panel was composed of **Shibadas Biswal,** MBBS, MD (Takeda), **Guillermo Herrera-Taracena,** MD, MBA (Johnson & Johnson), **Prasad Kulkarni,** MD (SII), **Louis Macareo,** MD, JD (Merck), and **Kirk Prutzman**, PhD (FDA). In this broad-ranging session, the panelists emphasized the following key points:

- Managing expectations and clearly communicating the development and deployment timelines for dengue countermeasures is critical for retaining and building public trust and acceptance of new vaccines and drugs. There is significant interest in new vaccines and therapeutics in many regions of the world—especially those experiencing severe dengue outbreaks—and it can be frustrating to both individual consumers and political leaders when promising interventions and/or countermeasures are not readily available. Clearly communicating the complexity of developing, licensing, and distributing new vaccines and therapeutics may help streamline both development and distribution efforts while retaining public enthusiasm and support for the interventions.
- The panelists also advocated for thinking ahead to how the next generation of dengue countermeasures will be evaluated for clinical safety and efficacy. With multiple DENV vaccines now licensed or nearing licensure there are new ethical and scientific considerations regarding the appropriate control groups for such studies. It may not be desirable from both a scientific and ethical perspective to utilize a placebo group for future vaccine studies, as the appropriate comparison group may be the current “standard of care/vaccination” should the uptake of DENV vaccination become more widespread.

## 13. Session 11: Reading between the lines: Supporting countermeasure development and deployment decisions with imperfect data

The first session of the third and final day of the summit was moderated by **Michael Johansson**, PhD (CDC, Northeastern University) and was focused on how advances in epidemiologic and immunologic models can inform dengue control policies and countermeasure deployment decisions in the absence of perfect data.

**Alex Perkins,** PhD (ND) gave the first presentation of the session, describing how epidemiological models support the assertion that the sustainable control of dengue will require coordinated deployment of multiple intervention strategies. This includes integrated deployment of sustainable vector control, vaccines, and infrastructure development. Dr. Perkins stressed that it has become increasingly clear over the last 60 years that there are no silver bullets for achieving dengue control. He posited that new mathematical models are needed to better understand what combination of interventions might best achieve the goal of dengue control in an affordable and sustainable fashion. He additionally stressed that the models required for accurately assessing the impact of these complex intervention strategies must (1) capture the long-term dynamics of the virus, vector, and host immunity, (2) accurately reflect the effects of different intervention strategies, (3) provide insight regarding the cost effectiveness of the proposed approaches, and (4) account for the diverse DENV epidemiologic profiles observed around the world. Accordingly, Dr. Perkins highlighted recent modeling efforts which demonstrated that even if existing vector control tools (spraying, *Wolbachia-*mediated vector replacement or suppression) are used in an optimal fashion, they can substantially reduce transmission in the short-term but are not capable of providing long-term dengue control due to the buildup of susceptibility that follows the success of those interventions. Models also demonstrate that current vaccines could help alleviate disease burden but may not offer substantial cost savings. Combining models that incorporate “building out” (a shorthand for improvements in urban infrastructure that reduce human–mosquito contact), the vector (e.g., improved housing structures), vaccination, and optimal use of existing vector control interventions resulted in predictions of sustainable dengue control. Building out could offer a long-term solution and would synergize well with near-term enhancement of control. Ongoing work is assessing the generalizability of these models and their conclusions across different transmission settings. An important drawback of these models is that they assume equal prevalence of all four serotypes. More data is necessary to be able to model according to varying prevalences of the four DENV serotypes. Altogether, this suggests that a multi-faceted approach leveraging short-term enhancements and long-term investments in resolving underlying problems is required for sustainable dengue control.

Next, **Ilaria Dorigatti**, PhD (Imperial College London) presented on the challenges and opportunities in modeling dengue interventions. Dr Dorigatti’s group focused on the analysis of data from the TAK-003 clinical trial to better characterize the efficacy profile of the Takeda dengue vaccine by modeling vaccine action and the relative risk of symptomatic disease and hospitalization as a function of neutralizing antibody titers. In turn, the efficacy profile of TAK-003 was used to assess the potential individual- and population-level impact of vaccination across transmission settings [78]. The developed models could reconstruct the number of cases reported in the vaccine and placebo arm over time against all four serotypes, where efficacy was generally predicted to be very high for DENV-2, and lower for DENV-1, 4, and 3 in this order. Notably, the vaccine efficacy estimates varied by baseline serostatus, where seronegative individuals had negative central vaccine efficacy estimates around 3 years post-vaccination reflecting a risk of disease enhancement upon DENV-3 and DENV-4 infection. Dr Dorigatti’s group also developed models to estimate the transmission intensity of dengue as measured by the force of infection, which can be translated into estimates of the seroprevalence at a specific age (e.g., at 9 years of age) that can be used to compare the risk of dengue infection across locations and assess the suitability of vaccination with the TAK-003 vaccine [79]. Dr Dorigatti presented the most recent global dengue transmission intensity and burden estimates - work which has been translated into an interactive web tool providing access to global estimates of dengue transmission intensity, seroprevalence, and burden, as requested by the WHO. Importantly, estimates of the force of infection in locations with no available epidemiological (age-stratified serological and/or case-notification) data were reconstructed using machine learning based on the local climate, environmental factors, and demography. A current assumption and key limitation of these models, however, is that they assume equal transmissibility of the four serotypes. There is a need for more serotype-specific serological data and case data to get serotype-specific estimates of transmission intensity. Another key limitation of the current risk maps is the limited available data on dengue and arboviruse transmission in Africa. To address this, Dr Dorigatti is working with international partners on the implementation of xSTAR—a multiplex Serology Testing and Analysis Platform that aims to develop a standardized and validated multiplex serology assay, and deliver training, which will promote testing, data analysis, and modeling in Africa. The objective is to validate this assay and expand its use to better understand arbovirus circulation, transmission, and immunity in Africa and globally.

### Key session insights and discussion points

- Dengue endgame does not necessarily mean eradication, but control.
- Disease modeling indicates that no single existing intervention (vaccine, vector control, *Wolbachia-*mediated vector replacement and suppression, environmental modification) can achieve control, and an integrated approach combining the use of multiple imperfect interventions is necessary to achieve dengue control.
- Benefit and costs of interventions vary in time and taking the time scale into consideration is important when performing prospective assessments on their potential impact at the population level.
- Short-term interventions can be helpful especially in outbreak contexts or specific settings.
- There is a need to collect serotype-specific serology and case-notification data to validate current assumptions on the transmissibility of the four dengue serotypes and/or generate evidence on serotype-specific transmission intensity estimates. This will allow improving current models and in turn the impact assessment of existing and new intervention strategies ahead of deployment.

## 14. Session 12: Vaccines alone don’t save lives... addressing vaccine hesitancy and scientific misinformation in support of a dengue endgame

The final session of the summit—moderated by **Rebecca Christofferson**, PhD (LSU)—had the objectives of discussing the challenges and opportunities associated with developing and communicating dengue-related policies and public-health information.

First, **Telisa Stewart,** MPH, DrPH (SUNY Upstate) began the conversation with a presentation entitled “*Communication and Behavioral Health Vaccination Playbook – Patients and Populations*”. In her presentation, Dr. Stewart posited that scientific and medical communication gaps persist across many aspects of society due to differing levels of scientific literacy and preconceived notions regarding roles and responsibilities related to the production, dissemination, and consumption of scientific information by the scientific community and the general public. Physicians and scientists have a key role in translating and disseminating scientific messages across the scientific community and the general public. Physicians and scientists remain trusted public figures to many individuals, and having an understanding of the principles of scientific communication can help improve the effectiveness of engagement across the general population and the scientific community. To this end, Dr. Stewart highlighted several fundamental concepts of effective scientific communication that should be considered when endeavoring to convey information in the public-health space. These include (1) understanding and clearly articulating the purpose of the communication, (2) understanding the most effective mechanism for communication delivery, (3) being aware of the context of the communication, and (4) understanding the intended audience. Each of these elements should be carefully considered to effectively convey information to a given audience. Avoiding scientific jargon and being aware of familiar/native language of a population is important. Awareness of the viewpoint of the audience you are trying to communicate to is key, especially regarding the cultural values, norms, and perceptions of health literacy. Finally, with ample sources of information, it is important to give audiences the ability to sift through misinformation and disinformation, to not only convey your message but provide appropriate resources for individuals to continue to explore for themselves and know how to identify credible sources.

This was followed by presentation by **Jana Shaw,** MD, MPH, MS (SUNY Upstate) on strategies to address vaccine hesitancy. Despite the increasing prevalence of disinformation and hesitancy regarding childhood vaccination in the United States, over 92% of children complete the recommended early childhood vaccination schedule prior to entering kindergarten [80]. However, clear geographic/regional differences in vaccine acceptance/uptake exist, providing an opportunity to understand the underlying causes of this phenomenon. Causes of vaccine hesitancy include broad dissemination of misinformation regarding the safety and efficacy of vaccines, distrust in institutions tasked with producing/approving/regulating/administering vaccines, cultural or religious beliefs, fear of adverse events following vaccination, and low perceived risk of the diseases targeted by the vaccines [81]. To effectively address false information and combat vaccine hesitancy, it is essential to employ evidence-based communication strategies that resonate with hesitant individuals. A key approach is the use of presumptive communication, which frames vaccination as the default and normal course of action [82]. Tailoring vaccine information to address an individual’s specific concerns is another critical component, as it demonstrates empathy and understanding. This can be achieved using the five-step motivational interviewing technique, which involves establishing empathy and credibility, briefly addressing specific concerns, emphasizing disease risk and vaccine effectiveness, and concluding with a strong, personalized recommendation [83]. In addition to one-on-one interactions, broader strategies include enhancing education and awareness among target populations through culturally sensitive outreach programs and accessible materials. Providing fact-checking resources—such as verified websites or mobile applications—helps individuals identify and counter misinformation. Furthermore, creating engaging platforms that promote vaccination, such as community forums, social media campaigns, or interactive workshops, can foster trust and facilitate informed decision-making. Together, these strategies create a cohesive, evidence-based framework to reduce hesitancy and encourage vaccine uptake. In particular, increasing trust in the healthcare system is a crucial and highly effective strategy for combating vaccine hesitancy and can be accomplished by fostering a culture of transparency, cultural competency, and community engagement. An integrated approach involving the collaboration of community leaders and healthcare organizations is often the most effective approach for understanding—and addressing—the unique viewpoints and perspective of vaccine-hesitant populations.

The final presentation of the summit was by **Hoe Nam Leong,** MD (Singapore), who presented on the history of dengue vaccines in Asia and how there is no escape from vaccine hesitancy in endemic areas. In the Philippines, vaccine hesitancy is on the rise due to previous issues with vaccine rollout and retraction due to increased risk of severe disease, which have caused hesitancy with regards to vaccine safety. There was a large amount of disinformation with respect to the impact of vaccination on severe disease risk and many of the children’s deaths in the Philippines were not properly evaluated. Looming Dengvaxia hesitancy proved to be further detrimental as it impacted COVID-19 vaccine uptake in the Philippines, as well as in a number of southeast Asian countries including Malaysia, Indonesia, and Vietnam. Studies have conducted surveys indicating factors that determine vaccine acceptance and hesitancy, with findings that can be leveraged to reinforce factors of vaccine acceptance and address factors associated with hesitancy. Importantly, practicing cultural humility is key for increasing vaccine uptake in hesitant regions, where community engagement by local leaders is essential for building trust.

### Key session insights and discussion points

- In vaccine hesitancy, complex determinants require a comprehensive approach.
- “Don’t just wing it”—build a multidisciplinary team, use the science to generate health messages and behavior change, and leverage the science on how information travels and on uptake behavior.
- Understanding the perspectives of hesitant populations is crucial to addressing hesitancy and tailoring approaches to improve trust.

### Key summit observations and calls for action

At the conclusion of the summit, the speakers and summit highlighted the following key observations and opportunities to directly advance toward the goal of sustainable and functional dengue control. Many of the key discussion points highlighted here and in **Table 1** have inspired the themes for the next sunnit which will be held in August 2025, including session on how to best define and categorize dengue sevierity, how dengue epideomological trends are changing over time, and the requirements for global leadership in order to achieve a true dengue endgame.

1. The absence of an immunologic marker that is statistically associated with protection from dengue virus infection or disease is a fundamental knowledge gap hindering countermeasure development. While it is unlikely that a single marker of protective immunity exists across all dengue infection scenarios (e.g., different dengue immune backgrounds, dengue virus types, spectrum of clinical outcomes, etc.), new methods of profiling innate and adaptive immunity prior to infection and characterization of immunologic, virologic, and clinical outcomes following infection offer new opportunities to define immune correlates of protection and pathogenesis.
2. There remains the need for a method(s) of classifying dengue disease severity across settings, which is largely objective, broadly applicable, unaffected by different standards of medical care practice, and is designed to support highly regulated clinical trials of dengue countermeasures such as vaccines, drugs, and other biologics. Standardizing a method(s) for classifying dengue disease severity is essential not only for efficacy determinations but for assessments of countermeasure safety (i.e., the potential a vaccine or other biologic could worsen infection outcomes compared to placebo or control).
3. Changing epidemiologic trends in the burden of dengue virus infections and disease severity (i.e., increasing incidence of dengue in adults, increasing incidence of severe dengue in primary infections, etc.) necessitates the reassessment of many fundamental hypotheses and understandings of DENV immunopathogenesis and manifestations of dengue disease. In addition, flavivirus immunologic cross-talk studies need to be extended beyond dengue-Zika infections and the impact of preexisting flavivirus immunity (both from infection and vaccination) needs to be evaluated comprehensively in target populations.
4. There is a pressing need for a significant expansion of the breadth and depth of dengue virus infection and disease surveillance using existing serologic and molecular laboratory techniques and the exploration of newer collection and testing techniques that offer the potential for increased access, efficiency, and cost effectiveness.
5. No single existing measure (vaccine, vector control, environmental modification, etc.) can achieve significant and sustained dengue control. An integrated approach combining optimal use of interventions is required.
6. The challenges with communicating complex scientific and medical information to the general public during the COVID-19 pandemic fueled anti-science and vaccine hesitancy and presented the potential that safe and effective dengue countermeasures may go unused. Scientists and clinicians need to pursue education and training in health communications.
7. Finally, dengue needs to become a global health priority again and requires strong global leadership across the multiple disciplines, domains, and institutions which will contribute to developing accessible and sustainable solutions and mitigations to the current dengue burden.
